# 

**Published:** 1855-05

**Authors:** 


### BUFFALO MEDICAL JOURNAL

AND

### Monthly Review

OF

### MEDICAL AND SURGICAL SCIENCE.

AUSTIN FLINT, M. D.,
SANFORD B. HUNT, M. D., } Editors.

BUFFALO, N. Y.:

PUBLISHED BY THOMAS & LATHROPS.

Commercial Advertiser Buildings.

1855.

Price $2.50 per annum, in advance.

